# Study protocol for a nationwide implementation of internet-based vestibular rehabilitation for patients with chronic vestibular symptoms (I-RECOVER)

**DOI:** 10.1186/s43058-023-00524-1

**Published:** 2023-11-22

**Authors:** Hà T. N. Ngo, Otto R. Maarsingh, Raymond van de Berg, Marco H. Blanker, Tjasse D. Bruintjes, René Castien, Rob Dijkstra, Sandra Rutgers, Pauline Slottje, Jos W. R. Twisk, Lucy Yardley, Jettie Bont, Vincent A. van Vugt

**Affiliations:** 1 Department of General Practice, Amsterdam UMC, Location Vrije Universiteit Amsterdam, Boelelaan 1117, Amsterdam, The Netherlands; 2 Amsterdam Public Health Research Institute, Amsterdam, The Netherlands; 3 Department of Otorhinolaryngology and Head and Neck Surgery, Maastricht University Medical Centre, Maastricht, The Netherlands; 4 Department of Primary and Long-Term Care, University Medical Centre Groningen, Hanzeplein 1, Groningen, The Netherlands; 5 Department of Otorhinolaryngology, Leiden University Medical Centre, Leiden, The Netherlands; 6 Department of Otorhinolaryngology, Gelre Hospital Apeldoorn, Apeldoorn, The Netherlands; 7 Faculty of Behavioural and Movement Sciences, Vrije Universiteit Amsterdam, Amsterdam Movement Sciences Program Musculoskeletal Health, Amsterdam, The Netherlands; 8 Patient Association Hoormij•NVVS, Houten, The Netherlands; 9 Department of Epidemiology and Biostatistics, Amsterdam UMC, Vrije Universiteit Amsterdam, Amsterdam Public Health, Amsterdam, The Netherlands; 10 School of Psychology, University of Southampton, Southampton, UK; 11 School of Psychological Science, University of Bristol, Bristol, UK

**Keywords:** Vestibular symptoms, Dizziness, Vertigo, Primary care, General practice, Vestibular rehabilitation, Implementation

## Abstract

**Background:**

Vestibular rehabilitation is a safe and effective exercise-based treatment for patients with chronic vestibular symptoms. However, it is underused in general practice. Internet-based vestibular rehabilitation (Vertigo Training), which has proven to be effective as well, was developed to increase uptake. We now aim to improve the quality of care for patients with vestibular symptoms by carrying out a nationwide implementation of Vertigo Training. We will evaluate the effect of this implementation on primary care.

**Methods:**

Our implementation study consists of three successive phases:

1) We will perform a retrospective observational cohort study and a qualitative interview study to evaluate the current management of patients with vestibular symptoms in primary care, in particular anti-vertigo drug prescriptions, and identify areas for improvement. We will use the results of this phase to tailor our implementation strategy to the needs of general practitioners (GPs) and patients.

2) This phase entails the implementation of Vertigo Training using a multicomponent implementation strategy, containing: guideline adaptations; marketing strategy; pharmacotherapeutic audit and feedback meetings; education; clinical decision support; and local champions.

3) In this phase, we will evaluate the effect of the implementation in three ways.

a. Interrupted time series. We will use routine primary care data from adult patients with vestibular symptoms to compare the number of GP consultations for vestibular symptoms, referrals for vestibular rehabilitation, prescriptions for anti-vertigo drugs, and referrals to physiotherapy and secondary care before and after implementation.

b. Prospective observational cohort study. We will extract data from Vertigo Training to investigate the usage and the characteristics of participants. We will also determine whether these characteristics are associated with successful treatment.

c. Qualitative interview study. We will conduct interviews with GPs to explore their experiences with the implementation.

**Discussion:**

This is one of the first studies to evaluate the effect of a nationwide implementation of an innovative treatment on Dutch primary care. Implementation strategies have been researched before, but it remains unclear which ones are the most effective and under what conditions. We therefore expect to gain relevant insights for future projects that aim to implement innovations in primary care.

**Supplementary Information:**

The online version contains supplementary material available at 10.1186/s43058-023-00524-1.

Contributions to the literature

- There is substantial literature on implementation strategies in primary care. However, it remains unclear which ones are the most effective.
- As one of the first studies to evaluate a nationwide implementation of an innovative treatment in Dutch primary care, we expect to provide relevant insights for future implementation projects.
- We will employ several research methods, including an interrupted time series, to evaluate the implementation. An interrupted time series is considered the strongest design when a randomised controlled trial is not feasible.
- By evaluating the effectiveness of the individual components of our implementation strategy, we can provide guidance to others in the development of strategies.

## Background

Vestibular symptoms, such as dizziness and vertigo, affect approximately 25% of the general population at some point in their lives [1]. These complaints are therefore frequently seen in general practice. In a significant number of patients with vestibular hypofunction, vestibular symptoms become persistent and they develop a chronic vestibular syndrome [2]. For instance, 30–40% of patients with vestibular neuritis still experience dizziness after 6 months [3]. One of the reasons includes failure of the vestibular compensation process, which involves adaptation of the nervous system to vestibular damage [4]. According to the International Classification of Vestibular Disorders, a chronic vestibular syndrome comprises chronic vestibular symptoms lasting months to years and includes features suggestive of persistent vestibular system dysfunction [5, 6]. The prevalence of chronic vestibular symptoms in the adult population ranges between 1.4 and 4.8% [7, 8].

Adequate treatment of patients with vestibular symptoms, and especially chronic vestibular symptoms, is of utmost importance because the consequences are far-reaching. Many patients experience a reduced quality of life. Older patients, for instance, may experience loneliness, isolation, and decreased self-esteem [3, 9]. Furthermore, vestibular symptoms are associated with a high financial burden as a result of increased healthcare utilisation, increased frequency of falls, and sick leave [8, 10].

Vestibular rehabilitation (VR) is an exercise-based treatment that stimulates vestibular compensation. It is proven to be a safe and effective treatment for both patients with unilateral vestibular hypofunction (e.g. vestibular neuritis), and bilateral vestibular hypofunction (e.g. vestibulopathy due to aminoglycoside ototoxicity) [11, 12]. In addition, a systematic review in 2018 showed that VR is effective in reducing symptoms in patients with chronic vestibular symptoms, regardless of the original cause [13]. Despite strong scientific evidence, VR is underused in general practice, mostly due to a lack of knowledge [14, 15]. In contrast, off-label anti-vertigo drugs are prescribed regularly, despite low quality of evidence [16–18].

Safe and effective self-help applications for VR have been developed to increase uptake in the United Kingdom and the Netherlands [19, 20]. In 2017 we developed Vertigo Training, a Dutch version of internet-based VR, and performed a large randomised controlled trial (RCT) [20]. Adults aged 50 years and older with chronic vestibular symptoms were randomised to stand-alone VR, blended VR (with physiotherapy support), and usual care. Patients in both the stand-alone group and blended VR group experienced a clinically relevant decrease in vestibular symptoms, dizziness-related impairment, anxiety, and depressive symptoms up to 36 months follow-up [21].

### Study aims and hypotheses

With this study, we aim to improve the quality of care for patients with chronic vestibular symptoms by carrying out a nationwide implementation of Vertigo Training, using a multicomponent implementation strategy. We hypothesise that the implementation will enhance knowledge on (the management of) chronic vestibular symptoms, in both healthcare providers and patients. As a result of this, VR referrals will increase and anti-vertigo drug prescriptions will decrease. Therefore, the first aim of this study is to determine the effect of the implementation on primary care for patients with vestibular symptoms. Our second aim is to gain more insight into the effectiveness of the individual components of our implementation strategy, as this might inform future implementation projects. After all, a systematic review in 2015 showed that it is still unclear which implementation strategies are most effective in primary care and under what conditions [22]. This protocol describes our implementation study in which we employ both quantitative and qualitative research methods.

## Methods

**Fig. 1:**
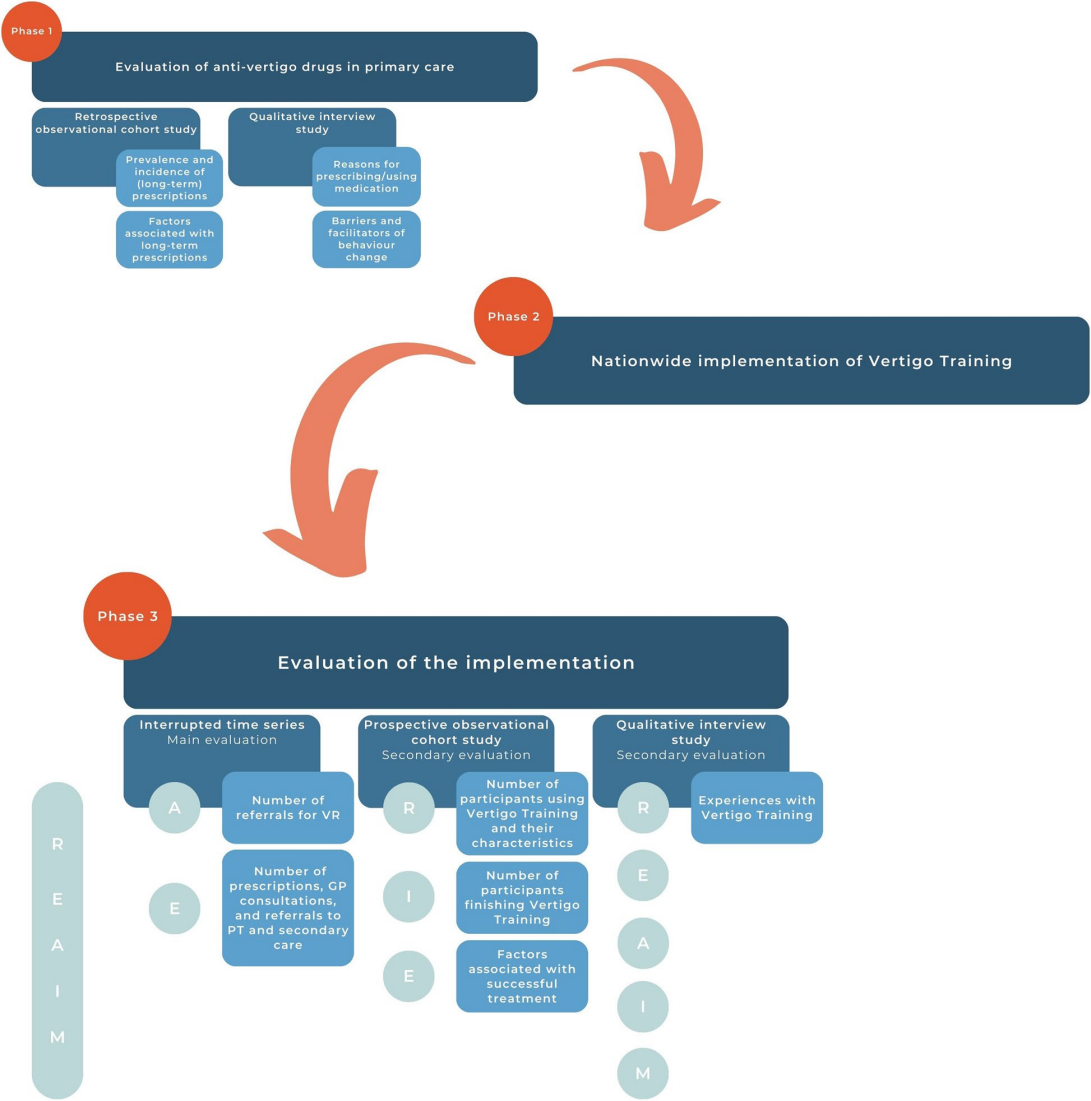
Overview of the implementation study including employed research methods and measured outcomes. *R* = Reach,* E* = Effectiveness,* A* = Adoption,* I* = Implementation,* M* = Maintenance,* GP* = general practitioner,* PT* = physiotherapist*, VR* = vestibular rehabilitation

This implementation study consists of three successive phases with different research methods. Phase 1 is a pre-implementation phase, phase 2 includes the implementation, and phase 3 entails the evaluation of the implementation. An overview of the phases including employed research methods and measured outcomes is shown in Fig. 1.

We used the RE-AIM (Reach, Effectiveness, Adoption, Implementation, Maintenance) framework, a frequently used framework for planning and evaluating implementation [23], to develop the multicomponent implementation strategy. Furthermore, we will apply this framework to evaluate the implementation both during the implementation period, in order to adapt the strategy accordingly, as well as after this period. The RE-AIM framework has been applied in over 450 publications, covering many different populations, settings, and health conditions [24].

## Phase 1: evaluation of anti-vertigo drugs in primary care

In this phase, we will evaluate the current management of patients with vestibular symptoms in primary care, in particular anti-vertigo drug prescriptions, and identify areas for improvement. We will use the results of this phase to tailor our implementation strategy to the needs of general practitioners (GPs) and patients.

### Retrospective observational cohort study

#### Study design and data collection

We reported this retrospective observational cohort study in line with the Strengthening the Reporting of Observational Studies in Epidemiology (STROBE) Statement (Additional file 1) [25]. We will evaluate prescribing practices for anti-vertigo drugs by investigating both the prevalence and incidence of (long-term) prescriptions and characteristics associated with long-term prescriptions.

We will use anonymised routine primary care data from more than 1.2 million patients registered at 269 general practices. In the Netherlands, almost all noninstitutionalised citizens are registered with one particular GP. The included general practices participate in the Intercity Collaboration [26], which comprises academic networks of general practice at the university medical centres of Amsterdam (Academic Network of General Practice Amsterdam UMC (ANHA)), Utrecht (Julius General Practitioners’ Network (JGPN)), Groningen (Academic General Practitioner Network Northern Netherlands (AHON)), and Maastricht (Research Network Family Medicine (RFNM)). These networks run ongoing longitudinal databases containing pseudonymised data extracted from electronic patient records of the participating general practices. The databases can conditionally be used for scientific research that is relevant for (primary) care. Patients are informed about this by their GP. The databases contain data of all registered patients, except for those who objected to this (opt-out).

For this study, data will be selected from patients, aged 18 years and older, with vestibular symptoms and/or prescriptions for anti-vertigo drugs from the period March 2018 to March 2021. Patients with vestibular symptoms will be identified with International Classification of Primary Care (ICPC) codes and patients with prescriptions for anti-vertigo drugs will be identified with Anatomical Therapeutic Chemical (ATC) codes. The list of used ICPC and ATC codes is detailed in Table 1.

**Table 1:** ICPC codes^a^ and ATC codes^b^ used for identification of patients and prescriptions

ICPC codes	ATC codes
N17 (Vertigo/dizziness)	N07CA01 (Betahistine)
N17.01 (Spinning dizziness)	N07CA02 (Cinnarizine)
N17.02 (Lightheadedness)	N07CA03 (Flunarizine)
H82 (Vertiginous syndrome)	N07CA52 (Cinnarizine, combinations)
H82.01 (Ménière’s disease)	
H82.02 (Vestibular neuritis)	
H82.03 (Benign paroxysmal positional vertigo)	

^a^International Classification of Primary Care codes

^b^Anatomical Therapeutic Chemical codes

We will collect data on demographic characteristics, registered ICPC codes for vestibular symptoms and comorbidity, prescriptions for anti-vertigo drugs, GP consultations for vestibular symptoms, and characteristics of the general practices (e.g. degree of urbanisation). Table 2 shows an overview of the variables, including those that are used for the interrupted time series (phase 3.1).

**Table 2:** Overview of variables for retrospective observational cohort study (phase 1) and interrupted time series (phase 3)

	**Variable**	**Definition**
**Patient characteristics**	Age	In years at time of registration ICPC code for vestibular symptoms
	Sex	Male or female
	Diagnosed vestibular disorder	According to ICPC code (see Table 1)
	Comorbidity	According to the following ICPC codes: K86 (Hypertension uncomplicated), K87 (Hypertension complicated), K83 (Heart valve disease), K84 (Heart disease other), K75 (Acute myocardial infarction), K76 (Ischaemic heart disease without angina), T90 (Diabetes), K89 (Transient cerebral ischaemia), K90 (Stroke/cerebrovascular accident), P74 (Anxiety disorder/anxiety state), P76 (Depressive disorder), A49.02 (Polypharmacy)
**General practice characteristics**	Registered patients	Total number of patients registered at practice per year and total number of adult patients (aged 18 years and over) registered at practice per year
	Employed general practitioners	Total number of general practitioners per year with patients registered to their name
	Degree of urbanisation	Based on 4-digit postal code: not/hardly urbanised (< 1.000 surrounding addresses), moderately urbanised (1.000–1.500 surrounding addresses), strongly urbanised (1.500–2.500 surrounding addresses), and extremely urbanised (> 2.500 surrounding addresses)
**Other**	Date of registration ICPC code for vestibular symptoms	-
	Date of registration and deregistration at general practice	-
	Referral for vestibular rehabilitation^a^	As described in electronic patient record
	Prescription for anti-vertigo drug	According to the ATC code (see Table 1)
	Date of prescription	-
	Referrals to physiotherapist, ENT specialist, neurologist, and/or geriatrician^a^	As described in electronic patient record
	GP consultations	Total number of GP consultations (all types of consultations, including telephone consultations and home visits) per patient per year for vestibular symptoms

^a^Only applicable to interrupted time series

*ATC code* Anatomical Therapeutic Chemical code,* GP* general practitioner, *ICPC code* International Classification of Primary Care code

#### Outcome measures

The main outcome is the prevalence of prescriptions for anti-vertigo drugs. Other outcomes are the incidence of prescriptions, prevalence of long-term prescriptions, and patient and/or general practice characteristics that are associated with long-term prescriptions.

The prevalence will be calculated as the total number of patients who received at least one (long-term) prescription divided by the average number of patients between March 2018 and March 2021. The incidence will be calculated as the total number of patients with new prescriptions divided by the total number of person years in the cohort per year. A prescription will be considered ‘new’ when a period of at least 6 months has passed between the last date of the prior prescription and the first date of the new prescription. Long-term prescriptions will be defined as prescriptions for 90 days or more in 12 months’ time.

#### Analysis

We will perform descriptive statistics to determine the characteristics of patients and general practices and to calculate the prevalence and incidence of (long-term) prescriptions. A multivariable logistic regression analysis will be carried out to identify characteristics associated with a long-term prescription. We will use IBM SPSS Statistics (version 28.0) for the analyses.

### Qualitative interview study

#### Study design and data collection

We reported this qualitative interview study using the consolidated criteria for reporting qualitative research (COREQ) (Additional file 2) [27]. We will identify barriers and facilitators of behaviour change, i.e. decreasing anti-vertigo drug use/prescriptions and increasing VR use/referrals, in GPs and patients with vestibular symptoms by conducting semi-structured interviews. We will conduct these interviews before start of the implementation in April 2023. Recruitment of GPs will take place through general practices that are connected to the ANHA, and through the professional network of the research group members. Patients will be recruited through the recruited GPs and the patient association (Hoormij•NVVS). We will inform GPs and patients who are interested and gather their characteristics for purposive sampling. For GPs, we will seek diversity in age, gender, years of practice, location of practice, size of practice, and prescribing behaviour for anti-vertigo drugs. For patients, we will seek diversity in age, gender, place of residence, education level, diagnosed vestibular disorder, and usage of anti-vertigo drugs.

We will obtain informed consent before the interview. Interviews will last approximately 30 min and will take place online, at the patients’ home/at the general practice, or at Amsterdam University Medical Centre. We will use a topic guide developed with the Theoretical Domains Framework and COM-B model [28, 29]. All interviews will be audio-recorded and transcribed verbatim. Interviews will be conducted until data sufficiency is reached.

#### Outcome measures

In the interviews with GPs, we aim to understand why they prescribe anti-vertigo drugs and what barriers and facilitators GPs may see when they stop prescribing anti-vertigo drugs.

In interviews with patients, our goal is to identify reasons for using anti-vertigo drugs and barriers and facilitators that may arise when they stop using these drugs.

#### Analysis

We will use a thematic analysis approach, according to Braun and Clarke, for the analysis of the transcripts [30]. Transcripts will be divided into fragments and codes will be assigned to these fragments by two independent investigators. After the first few interviews, codes will be discussed by the two investigators and, when consensus is reached about the coding scheme, the remaining interviews will be coded. Subsequently, the investigators will identify themes and codes will be assigned to these themes. In the final stage, themes from different transcripts will be compared and we will search for relations. All findings will be discussed with the research group. To facilitate the analysis, MaxQDA 2022 will be used.

## Phase 2: nationwide implementation of Vertigo training

We will embed Vertigo Training in Dutch primary care from April 2023 onwards. The online application will be made freely available through the website www.gezondheidsmeter.nl. More information on Vertigo Training is described in Table 3.

**Table 3:** Information on intervention to be implemented: Vertigo Training

Vertigo Training is a freely accessible internet-based intervention that contains six exercises. These six exercises include eye, head, and body movements that aim to stimulate vestibular compensation, a process in which the nervous system reorganises and is able to overcome damage of the vestibular system. In addition, a form of exposure-based behavioural therapy takes place by deliberately provoking vestibular symptoms in a controlled environment. The application prescribes a personal exercise regimen based on the severity of symptoms while performing the exercises. Patients are asked to perform the exercises twice a day for 10 min. The regimen lasts 6 weeks. Patients can start with Vertigo Training on their own initiative, or they can be referred to the treatment by a healthcare provider. They can perform the exercises by themselves, but it is also possible to receive support from a healthcare provider, such as a physiotherapist. In the Netherlands, patients can access physiotherapy through self-referrals.
Patients are eligible for Vertigo Training if they are 18 years and older, if they have had vestibular symptoms for at least one month during most days of the week, and if these symptoms are provoked or exacerbated by head movements or visual stimuli.

We created an implementation plan, involved local implementation specialists, and identified relevant stakeholders (i.e. GPs, ENT specialists, physiotherapists, and patients with vestibular symptoms) to adapt our implementation strategy to their needs. The final strategy consists of the following components: guideline adaptations; mass and target marketing strategy; pharmacotherapeutic audit and feedback meetings (PTAMs); education for GPs, patients, and physiotherapists; clinical decision support; and local champions. We will elaborate on these components further on.

We used the Standards for Reporting Implementation Studies (StaRI) Statement to report the implementation strategy and the intervention (Additional file 3) [31].

### Multicomponent implementation strategy

The strategy contains the following components:

- Guideline adaptations
  The Dutch College of General Practitioners’ clinical guideline for vestibular symptoms will include a recommendation for internet-based VR together with a hyperlink to Vertigo Training. In addition, a hyperlink to this intervention will be included in regional transmural agreements.
- Mass and target marketing strategy
  We will generate national attention for chronic vestibular symptoms and the intervention using mass media (e.g. press releases, newspapers, radio and television shows, and social media). To reach healthcare providers, we will collaborate with Dutch medical journals. We will also collaborate with the patient platform Thuisarts.nl, the most popular visited medical website by patients nationwide [32]. This platform will inform patients about VR, provide instructions for the exercises, and add a hyperlink to Vertigo Training. Furthermore, the patient association will actively inform their members about the availability of the intervention. Lastly, we have developed an infographic that contains information on Vertigo Training. GPs can use this infographic in their waiting room and consulting room to maximise exposure to patients and as a reminder to the availability of the intervention. This infographic will be disseminated through media channels and along with the guideline for PTAMs.
- Pharmacotherapeutic audit and feedback meetings
  We will provide guidelines for PTAMs as a way to encourage GPs to stop prescribing anti-vertigo drugs and start referring patients to (internet-based) VR. In the Netherlands, these meetings are regularly organised by pharmacists and GPs in order to improve evidence based prescribing behaviour. GPs will see how they perform compared to their peers using anonymised prescription data extracted from electronic patient records. We will actively promote the PTAM guidelines to general practices connected to the academic general practice networks.
- Education for GPs, patients, and physiotherapists
  We will offer free education to GPs (in training), patients, and physiotherapists (in training) to improve their knowledge on chronic vestibular symptoms and VR. For instance, we will develop an e-learning module and host webinars.
- Clinical decision support
  We aim to add automatic recommendations for (internet-based) VR to electronic patient records. This recommendation would show up when a GP registers a vestibular ICPC code as a way to support them in choosing VR (instead of anti-vertigo drugs).
- Local champions
  We aim to recruit GPs from different regions throughout the Netherlands to act as local champions. Their role will be to encourage colleagues to use (internet-based) VR and to organise PTAMs. Furthermore, we will motivate local champions to start their own additional implementation activities.

## Phase 3: Evaluation of the implementation

The evaluation will take place in three ways:

### Interrupted time series

#### Study design and data collection

An interrupted time series compares longitudinal data before and after an intervention. This design can be used when a RCT is not affordable or possible and is especially useful for evaluating interventions in real world settings, such as in our case [33]. Therefore, this design has been widely used for evaluating changes in health policy and interventions that aim to improve health system quality [33, 34].

For this part of the study, we will also use anonymised routine primary care data from the Intercity Collaboration as described in the retrospective observational cohort study from phase 1. In this case, we will compare data from adult patients with vestibular symptoms and/or prescriptions for anti-vertigo drugs from the period before (April 2021–March 2023) and after (April 2023–March 2025) implementation to determine whether the implementation changed how GPs treat patients with vestibular symptoms and to explore the effectiveness of the individual components of our strategy.

Patients with vestibular symptoms and/or prescriptions for anti-vertigo drugs will be identified using ICPC codes and ATC codes that are detailed in Table 1. We will gather data on demographic characteristics, registered ICPC codes for vestibular symptoms and comorbidity, prescriptions for anti-vertigo drugs, GP consultations for vestibular symptoms, referrals for VR, and referrals to physiotherapy and secondary care. We will also gather information on the characteristics of the general practices. An overview of variables, including their definitions, can be found in Table 2.

#### Outcome measures

The primary outcome is the number of referrals for VR per 1000 patients per month (Adoption). To assess this outcome, we will review free text annotations. Two investigators will review a random sample of free text annotations to determine which terms are used for the referrals. We will use these terms to identify referrals for VR in patients with vestibular symptoms.

Secondary outcomes are the number of prescriptions for anti-vertigo drugs, GP consultations for vestibular symptoms, and referrals to physiotherapists, ENT specialists, neurologists, and geriatricians per 1000 patients per month (Effectiveness).

#### Analysis

**Fig. 2:**
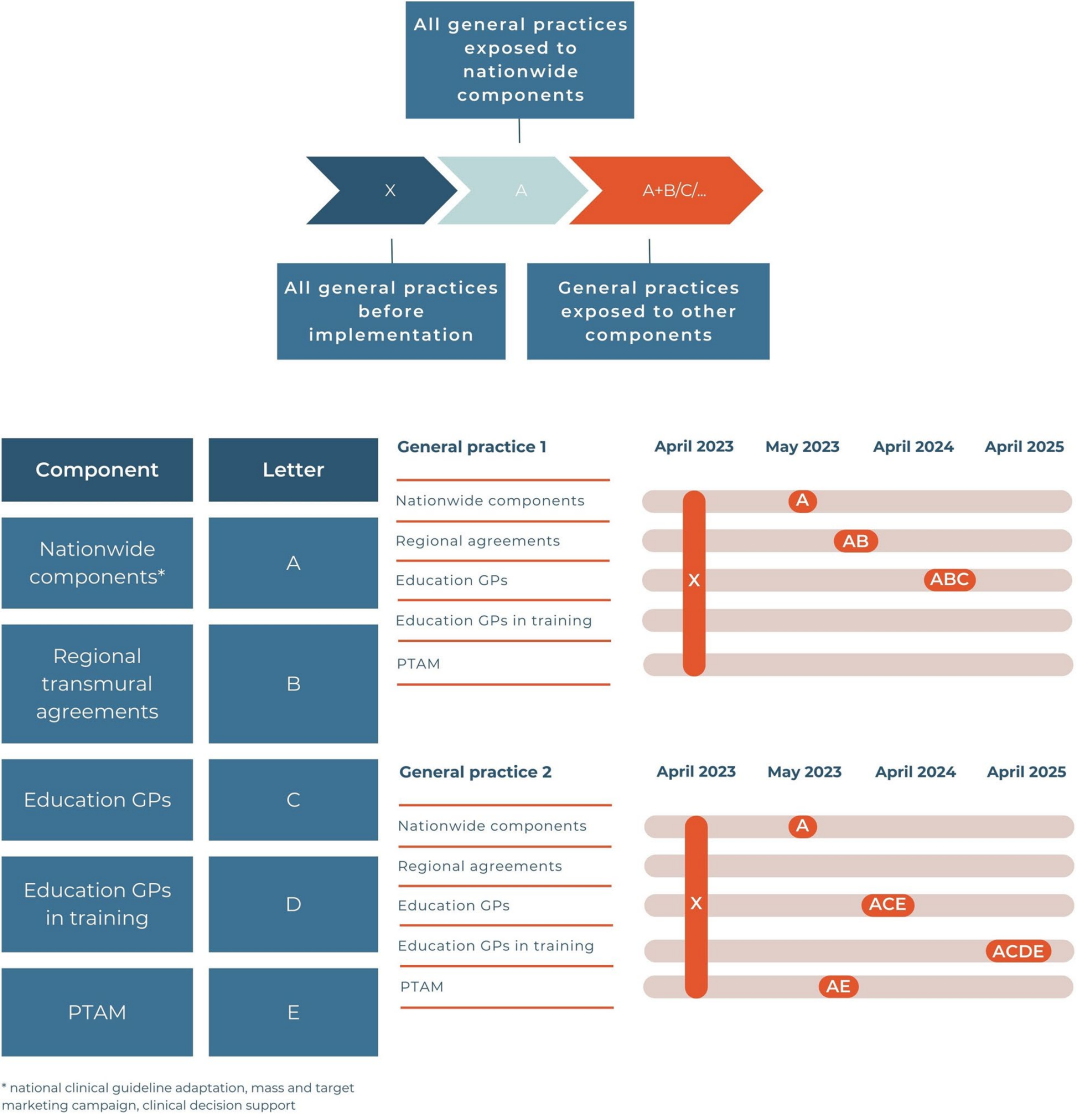
Example of measuring the effectiveness of the individual components. *GPs* = general practitioners, *PTAM* = pharmacotherapeutic audit and feedback meeting

We will analyse the number of patients with referrals for VR, prescriptions for anti-vertigo drugs, GP consultations for vestibular symptoms, and referrals to physiotherapists and secondary care using descriptive statistics. We will use monthly measurements in the period before (April 2021–March 2023; 24 data points) and after (April 2023–March 2025; 24 data points) implementation for the interrupted time series. A segmented regression analysis will be performed to compare outcomes between these two periods. This analysis uses statistical models to estimate the level and trend in the pre-intervention segment and changes in level and trend after the intervention [35]. By doing so, we can evaluate the overall effect of the implementation. To explore the effectiveness of the individual components of our strategy, we will compare changes in levels and trends between general practices that have been exposed to that specific component and general practices that have not been exposed. Figure 2 shows an example of how we are going to assess the effectiveness of the individual components. Descriptive statistics will be performed with IBM SPSS Statistics (version 28.0) and the segmented regression analysis will be performed with R software (version 4.2.1).

### Prospective observational cohort study

#### Study design and data collection

We reported this prospective observational cohort study in line with the The Strengthening the Reporting of Observational Studies in Epidemiology (STROBE) Statement (Additional file 4) [25]. During the implementation period (April 2023–March 2025), we will gather data from participants who use Vertigo Training and who give informed consent for the collection and analysis of their data. Participants who do not give informed consent can still use the application. We will evaluate the usage of Vertigo Training and the characteristics of participants. The evaluation will take place on a regular basis and we will adapt our implementation strategy accordingly. We will also determine whether certain characteristics are associated with successful treatment.

Data on the usage of Vertigo Training will be collected. We will also gather data on demographic characteristics, diagnosis and treatment of vestibular symptoms, and severity of vestibular symptoms according to the vertigo symptom scale-short form (VSS-SF) [36], using an online voluntary questionnaire at baseline. The VSS-SF will additionally be evaluated at 6 weeks, 3 months and 6 months. Table 4 shows an overview of variables.

**Table 4:** Overview of variables for prospective observational cohort study

Variable	Definition
Age	In years at time of start intervention
Gender	Male, female, other
Place of residence	-
Diagnosed vestibular disorder	Anamnestic
Education level	Primary education, prevocational secondary education, senior general secondary education, pre-university education, senior secondary vocational education, higher vocational education and university education
Migration background	Yes (if at least one parent was born abroad) or no
Received medical care for vestibular symptoms	Yes (from general practitioner, medical doctor at hospital, medical doctor at other type of clinic, physiotherapist, and/or other healthcare provider) or no
Prescription for anti-vertigo drug in past	Yes or no
Vestibular symptoms	According to vertigo symptom scale-short form at baseline, 6 weeks, 3 months, and 6 months
Acquaintance with Vertigo Training	Through the patient association, the internet, a healthcare provider, friends, social media, and/or other ways

#### Outcome measures

Outcomes are the number of participants using Vertigo Training (Reach), the number of participants finishing the exercise regimen (Implementation), and the descriptive characteristics of these participants (Reach). We will also evaluate characteristics associated with successful treatment (Effectiveness). Successful treatment will be defined as a change of 3 points or more in the VSS-SF score between baseline and 3 months, which indicates a clinically relevant difference in vestibular symptoms.

#### Analysis

Descriptive statistics will be used to assess the usage and characteristics of participants. In addition, we will perform a mixed-effects logistic regression analysis to evaluate whether these characteristics are associated with successful treatment. We will use IBM SPSS Statistics (version 28.0) for the descriptive statistics and the mixed-effects logistic regression analysis will be performed with R software (version 4.2.1).

### Qualitative interview study

#### Study design and data collection

We reported this qualitative interview study using the consolidated criteria for reporting qualitative research (COREQ) (Additional file 5) [27]. Semi-structured interviews with GPs will be conducted after the implementation period (from April 2025 onwards) to explore their experiences with the implementation and with Vertigo Training. We will also assess whether they intend to start and/or keep referring patients to the intervention.

We will recruit GPs through the academic general practice networks and contact GPs who gave permission to be approached for future research during the qualitative interview study from phase 1. We will inform GPs who are interested and gather their characteristics. Purposive sampling will be used, seeking diversity in age, gender, years of practice, location of practice, size of practice, degree of exposure to components of our strategy, and referral behaviour for VR.

Informed consent will be obtained before the interview. Interviews will last approximately 30 min and will take place online, at the general practice, or at Amsterdam University Medical Centre. We will use a topic guide that is developed with the RE-AIM framework [37]. We will audio-record all interviews and transcribe them verbatim. We will conduct interviews until we reach data sufficiency.

### Outcome measures

We will explore all dimensions of the RE-AIM framework (Reach, Effectiveness, Adoption, Implementation, Maintenance) during the interviews.

### Analysis

We will apply the same approach as during the qualitative interview study from phase 1.

## Discussion

Vestibular rehabilitation, a safe and effective exercise-based treatment for patients with vestibular hypofunction [11–13], is underused in Dutch general practice. Anti-vertigo drugs, however, are still regularly prescribed despite a lack of evidence base [16, 18]. Internet-based VR, which has shown to be safe and effective as well [19, 20], was developed to increase uptake. This implementation study aims to improve care for patients with vestibular symptoms by implementing Vertigo Training, a Dutch version of internet-based VR, nationwide using a multicomponent implementation strategy. We will evaluate the effect of the implementation on primary care and explore the effectiveness of the individual components of our strategy.

The main strength of this study lies in its employed research methods. For the evaluation of the implementation, we will carry out both an interrupted time series and a prospective observational cohort study. An interrupted time series is considered the strongest design to evaluate the effect of an intervention when a RCT is not feasible [33]. By additionally employing a qualitative research method, we can enrich the evidence. Furthermore, by regularly analysing prospective data from Vertigo Training and identifying barriers and facilitators, we can tailor our implementation strategy and increase the effectiveness of our implementation. Lastly, we will use routine primary care data from more than 1.2 million patients registered at general practices throughout the Netherlands in addition to data from participants using Vertigo Training. This supports the generalisability of our results. There are also methodological limitations to this study. As the effectiveness of the individual components of our strategy will be evaluated with an interrupted time series on general practice level, we have to make several assumptions. For instance, when one GP from a general practice with multiple GPs will attend a PTAM, we assume that this GP will have an impact on all patients who are registered at that general practice. This will have an influence on the estimation of the effectiveness. In addition, it will not be feasible to determine for all components whether or not general practices are exposed. We will, for example, not be able to separate who has been exposed to the mass marketing strategy and who has not been exposed. We are therefore restricted in measuring whether exposure to a specific component resulted in a change in outcomes. Furthermore, as patients can also perform the exercises using the patient platform Thuisarts.nl, it may be possible that only a selected group (e.g. patients with advanced digital skills) will use Vertigo Training, introducing bias into our data. We will not be able to gather data from patients using Thuisarts.nl to increase the validity of our results. Another consequence of this is that we cannot tailor our strategy to the needs of this specific patient group. The same accounts for patients with vestibular symptoms that would have an indication for Vertigo Training, but were not referred to the treatment and/or did not start the treatment. Lastly, Vertigo Training has broad inclusion criteria. Therefore, some participants might benefit less from the intervention than others. For example, the Epley manoeuver is more effective in patients with benign paroxysmal positional vertigo than VR in the short term [11]. This might influence the estimation of the effect of Vertigo Training.

As far as we know, this is one of the first studies to evaluate the effect of a nationwide implementation of an innovative treatment on Dutch primary care. Implementation strategies have been researched before, but it remains unclear which ones are the most effective and under what conditions [22]. We therefore expect to gain relevant insights for future projects that aim to implement innovations in primary care.

### Supplementary Information

**Additional file 1.**

**Additional file 2.**

**Additional file 3.**

**Additional file 4.**

**Additional file 5.**

## Data Availability

Not applicable.

## Abbreviations

ANHA: Academic Network of General Practice Amsterdam UMC
AHON: Academic General Practitioner Network Northern Netherlands
ATC: Anatomical Therapeutic Chemical
ICPC: International Classification of Primary Care
JGPN: Julius General Practitioners’ Network
GP: General practitioner
PTAM: Pharmacotherapeutic audit and feedback meeting
RCT: Randomised controlled trial
RE-AIM: Reach, Effectiveness, Adoption, Implementation, Maintenance
RNFM: Research Network Family Medicine
VR: Vestibular rehabilitation
VSS-SF: Vertigo symptom scale-short form
